# The CD8 Antiviral Factor (CAF) can suppress HIV-1 transcription from the Long Terminal Repeat (LTR) promoter in the absence of elements upstream of the CATATAA box

**DOI:** 10.1186/1743-422X-11-130

**Published:** 2014-07-21

**Authors:** Varsha Shridhar, Yue Chen, Phalguni Gupta

**Affiliations:** 1 Currently at the Department of Obstetrics and Gynecology, Magee Women’s Research Institute, University of Pittsburgh, Pittsburgh, USA; 2 Pittsburgh Retrovirology Laboratory, Department of Infectious Diseases and Microbiology, University of Pittsburgh Graduate School of Public Health, 426, Parran Hall, 130, DeSoto Street, Pittsburgh 15261, USA

**Keywords:** CD8 antiviral factor, HIV-1, Long Terminal Repeat, Transcription suppression

## Abstract

**Background:**

The CD8 Antiviral Factor (CAF) suppresses viral transcription from the HIV-1 Long Terminal Repeat (LTR) promoter in a non-cytolytic manner. However, the region on the LTR upon which CAF acts is unknown. Our objective was to determine the region on the LTR upon which CAF acts to suppress HIV-1 transcription.

**Methods:**

Serial deletions of the LTR from the 5’ end and inactivating point mutations were made.

**Results:**

Serial deletions of the LTR from the 5’ end indicated the importance of a short ~120 bp segment, containing the 3 SpI sites, CATA box (used by HIV-1 instead of the TATA box) and TAR region, in the suppressive process. Introduction of deletions or inactivating point mutations in the SpI sites or deletion of the TAR region did not abolish CAF-mediated transcriptional suppression. Yet, CAF-mediated transcriptional suppression was still retained in the HIV-1 CATA-TAR segment.

**Conclusion:**

CAF is able to suppress transcription from the LTR lacking all the elements upstream of the CATA box. Our results suggest that the HIV-1 CATA box may be responsible for CAF-mediated suppression of transcription from the HIV-1 LTR.

## Introduction

CD8^+^ T cells can control HIV-1 replication by non-cytolytic mechanisms [1,2]. The first non-cytolytic antiviral CD8^+^ T cell response was described in Long Term Non-Progressers of HIV-1 infection [1] and the factor mediating it was termed “CD8 Antiviral Factor” (CAF) [3]. CAF-mediated antiviral response has several characteristics: First, CAF suppresses HIV-1 mRNA production [4-8]. Second, CAF activity is not MHC-restricted or does not require direct contact between the CD8^+^ T cell and the target cell [9,10]. Third, CAF has been found to be effective against a wide range of HIV-1 clades, as well as HIV-2 and SIV and its activity inversely correlates with the stage of disease [11-18], and finally, both HIV-1 R5 and X4 viruses can be equally well suppressed [19-21]. Although many non-cytolytic CD8^+^ T cell factors have since been described [2,22-24], the identity and mechanism of action of CAF are as yet unknown.

The aim of this study was to elucidate the mechanism by which CAF mediates its HIV-1 transcription-suppressing effects. We hypothesized that CAF acts on and induces changes in the viral promoter to suppress transcription. Towards this, we focused on determining the region in viral promoter that was crucial for the suppressive effect of CAF. We performed serial progressive deletions on the LTR at 5’ end to identify the minimal region required for CAF-mediated transcriptional suppression. By a process of eliminating likely candidates, our data suggest that the HIV-1 CATA box (used by HIV-1 instead of the TATA box, motif: CATATAA, ref [25]) is the target for transcriptional suppression by CAF.

## Results and discussion

### CAF is HIV-1 LTR specific

**Figure 1:**
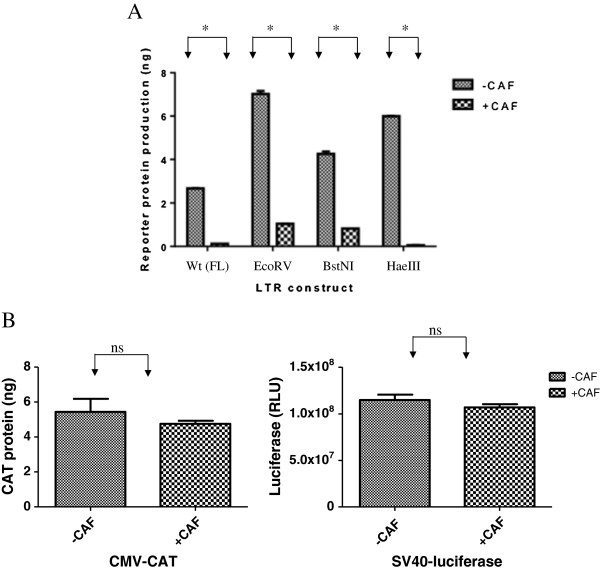
**Transcriptional suppression of HIV-1 LTR deletion mutants by CAF.** Reporter gene product expression in cells pretreated with Hanks Buffer (-CAF) or conditioned culture medium from a transformed CD8+ T cell line (+CAF) and transfected with wild type full-length (wt (FL)) HIV-1 LTR-CAT (Panel A) or CMV-CAT **(Panel B, Left )** or SV40-luciferase **(Panel B, Right)**. Results are representative from at least 2 independent experiments conducted in triplicate. *, p < 0.05; ns, not significant.

CAF has been shown to reduce the amount of HIV mRNA in infected CD4+ T cells [5]. We first sought to determine if the action of CAF was specific to a transfected HIV-1 LTR plasmid or if CAF could also act upon other transfected viral promoters. When 293 T cells, treated with CAF from the CD8^+^ T cells of an HIV-1 infected individual or with media control, were transfected with CMV-CAT (CAT = chloramphenicol acetyltransferase, a reporter gene), SV40-luciferase or HIV LTR-CAT constructs, we found that CAF was able to completely suppress reporter protein production in cell transfected with the full length HIV-1 LTR construct (referred to as “wt (FL)” in the figure) (Figure 1A). However, CAF did not suppress reporter-protein production in cells transfected with the CMV or SV40 constructs (Figure 1B). In all cases, MTT assays showed no cell death in CAF-treated and control cells (data not shown).

**Figure 2:**
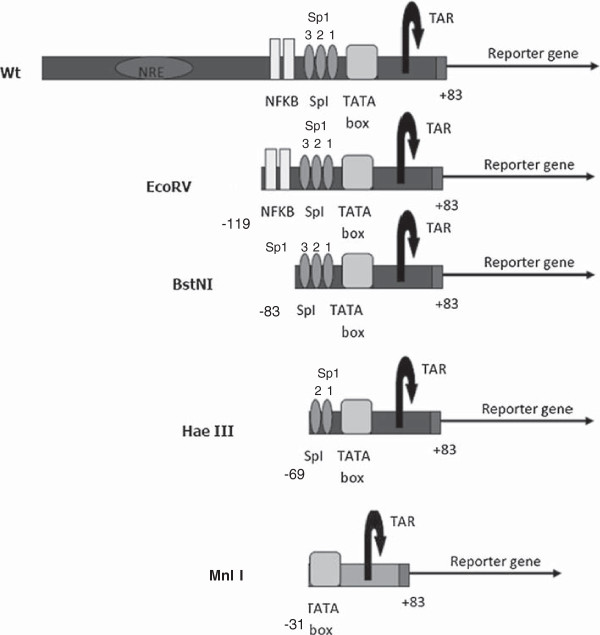
**Schematic of the HIV-1 Long Terminal Repeat (LTR) promoter showing the main transcription regulatory sites.** NRE, Negative Regulatory Region, NFKB, SpI sites, TATA box and TAR. EcoRV (-119), BstNI (-83), HaeIII (-69) and MnlI (-31) refer to the positions of the restriction enzyme site used to cleave the Wt full length LTR.

Thus, CAF seems to be specific to HIV. CAF might act by changing protein-DNA interactions or make epigenetic modifications to the HIV-1 LTR promoter. A previous paper [26] examined the effect of deletions and inactivating point mutations in certain regulatory elements and transcription factor binding sites on the HIV-1 LTR on the ability of CAF to suppress viral transcription. Their results showed that the replication of full-length HIV-1 molecular clones bearing mutations and deletions in individual transcription factor binding sites could still be suppressed in the presence of CAF. However, the authors did not study the effect of abolishing more than 1 promoter element simultaneously. We explored the possibility that more than one transcription factor-binding site or regulatory element on the viral promoter might be simultaneously involved in the process of CAF-mediated HIV-1 transcriptional suppression. The main segments of the LTR studied were the Negative Regulatory Element (NRE), the 2 NFKB sites, the 3SpI sites, and the CATA box in the U3 region, and the TAR bulge-and-loop, present in the R region (Figure 2). CAF might also function by making epigenetic modifications to the LTR. However, in this study, we did not examine the epigenetic profile of the LTR; rather we focused only on the LTR DNA.

### LTR progressive deletion constructs narrow the region of the promoter needed for CAF action

We used constructs that had been deleted of these regions from the HIV-1 LTR to investigate the possibility of multiple transcription factor (TF)-binding sites acting in conjunction with each other to suppress transcription in response to CAF. These constructs have been described before [27] and include the full length Wt-LTR, the EcoRV construct (containing deletion of the NRE), the BstNI construct (containing deletions up to the SpI sites), and the HaeIII construct (deletions up to the 3^rd^ Sp1 site and containing the 2 SpI sites most proximal to the CATA box, the TATA box and the TAR region). They were transfected into 293 T cells treated with either CAF or media control, and then stimulated with PMA. We found that CAF was able to suppress reporter gene expression from all of these constructs (Figure 1A). Sequential deletions of the LTR actually had higher transcriptional rates compared to the full-length promoter. This might be because of removal of negative regulatory regions from the LTR, leading to higher basal transcription in the absence of CAF.

These results indicate that CAF-mediated suppression of transcription is specific to HIV-1, is not affected by the type of reporter gene, and that the region encompassing the -69 to +83 of the LTR, containing 3 SpI sites, CATA box and the TAR region, is sufficient for the suppressive action of CAF.

### Role of the TAR loop in conferring susceptibility to CAF

The TAR region is a very attractive candidate target for CAF action. It has a unique bulge-loop structure and is well-conserved across all clades of HIV-1 [28]. Critical interactions between the TAR and the viral transactivator protein Tat take place on the bulge-loop structure on TAR [29,30]. If TAR were the target for CAF, it would help explain the specificity of CAF for HIV. Previous investigations on the region of the LTR necessary for CAF action probed the role of TAR by introducing inactivating, point mutations to disrupt the Tat-TAR interaction axis [26]. But if the structure of TAR, and not its sequence, were important for CAF action, point mutations might not indicate the importance of TAR in suppression.

**Figure 3:**
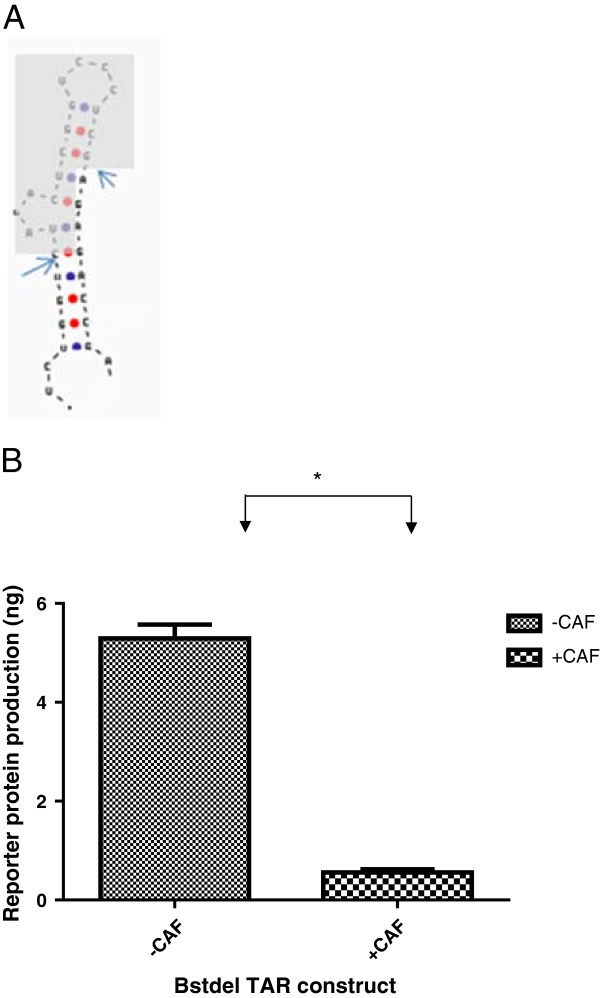
**Role of TAR in CAF suppression of HIV-1 LTR.** Schematic of the TAR from HIV-1 LTR with the arrows indicating the sites at which it was cleaved, and the shadow, the intervening region that was deleted **(Panel A)**. Reporter gene product (CAT) expression in cells pretreated with Hanks buffer (-CAF) or conditioned culture medium from a transformed CD8+ T cell line (+CAF) and transfected with the BstdelTAR construct, containing 3 SpI sites and TATA box **(Panel B)** Results are representative from at least 2 independent experiments conducted in triplicate. *, p < 0.05; ns, not significant.

For further scrutiny of the TAR, we selected the BstNI construct, which contains all the 3 SpI sites, HIV-1 CATA box and TAR regions. We deleted the bulge-loop region of the TAR (shaded in Figure 3A) from the BstNI construct, to form a new construct called BstΔTAR, to evaluate any changes in susceptibility to CAF. We found that, contrary to previous studies showing that the deletion of TAR drastically reduces the transcriptional ability of LTR [31,32], the TAR deleted BstΔTAR construct was still transcriptionally active and moreover, was suppressed by CAF (Figure 3B).

### Role of the SpI sites in CAF-mediated suppression

**Figure 4:**
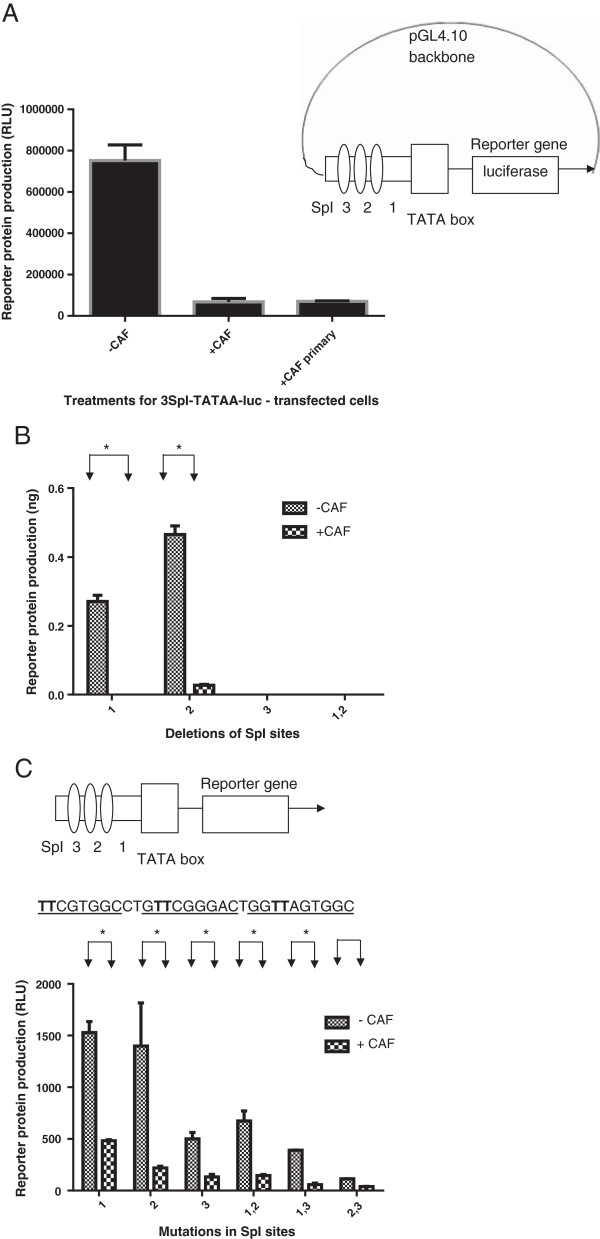
**Role of Sp1 sites in CAF suppression of transcription of HIV-1 LTR.** Suppression of reporter gene product (luciferase) expression in cells pretreated with Hanks buffer (-CAF) or conditioned culture medium from a transformed CD8+ T cell line (+CAF) and transfected with a construct containing only the 3Sp1 sites and TATA box of HIV-1 LTR. **(Panel A)** Reporter gene product (CAT) expression in cells pretreated with Hanks buffer (-CAF) or conditioned culture medium from a transformed CD8+ T cell line (+CAF) and transfected with Sp1 deletion constructs **(Panel B)** or SpI mutated constructs **(Panel C)**. The position of the specific Sp1 either deleted or mutated is given by the number on the x axis. The sequence in **Panel C** shows the bases (in bold) that were mutated to inactivate the SpI sites (underlined). CAF-primary refers to conditioned culture media from primary CD8+ T cells from an HIV infected individual Results are representative from 2 independent experiments conducted in triplicate. *, p < 0.05.

We then sought to determine if the 3 SpI-CATA segment of HIV-1 LTR could independently transcribe and get suppressed in response to CAF, in the absence of any other enhancer or promoter elements or the TAR sequence. Hence, we inserted the 3 SpI-CATA segment, which constitutes the HIV-1 minimal promoter, upstream of the luciferase reporter gene in a promoter-less vector, pGL4.10 and determined if it could independently transcribe. We found that the resultant construct could undergo LTR-driven transcription in response to PMA. Addition of CAF from transformed CD8^+^ T cell culture supernatants as well as CAF from primary CD8^+^ T cells from an HIV-1 infected individual (referred to as CAF_primary_) suppressed transcription from this construct efficiently (Figure 4A).Next, we deleted each SpI site individually or in pairs, in the absence of TAR, to check for changes in response to CAF. We found that deletion of the different SpI sites had different effects on LTR-driven transcription (Figure 4B). Deletion of SpI (1) or (2) still retained transcription, while deletion of SpI (3) or both (1) and (2) abolished transcription. However, as long as any construct was able to transcribe, it was suppressed by CAF. Since suppression of transcription is inextricably linked with the ability of the construct to transcribe, it was not possible to measure suppression in those constructs that were transcriptionally inactive.

To confirm that the results we observed were a product of SpI site inactivation and not because of the deletion process itself, we next inactivated the SpI sites by point mutation. Inactivation of the SpI sites, either individually or in pairs, resulted when key G residues in the SpI binding sites were replaced with T. Mutation of the SpI sites did not transcriptionally inactivate the construct, in accordance with previous reports [33]. While mutation of the various SpI sites was seen to affect transcription, all constructs were able to get suppressed in response to CAF (Figure 4C).

### Role of the CATA-box in CAF mediated HIV-1 suppression

**Figure 5:**
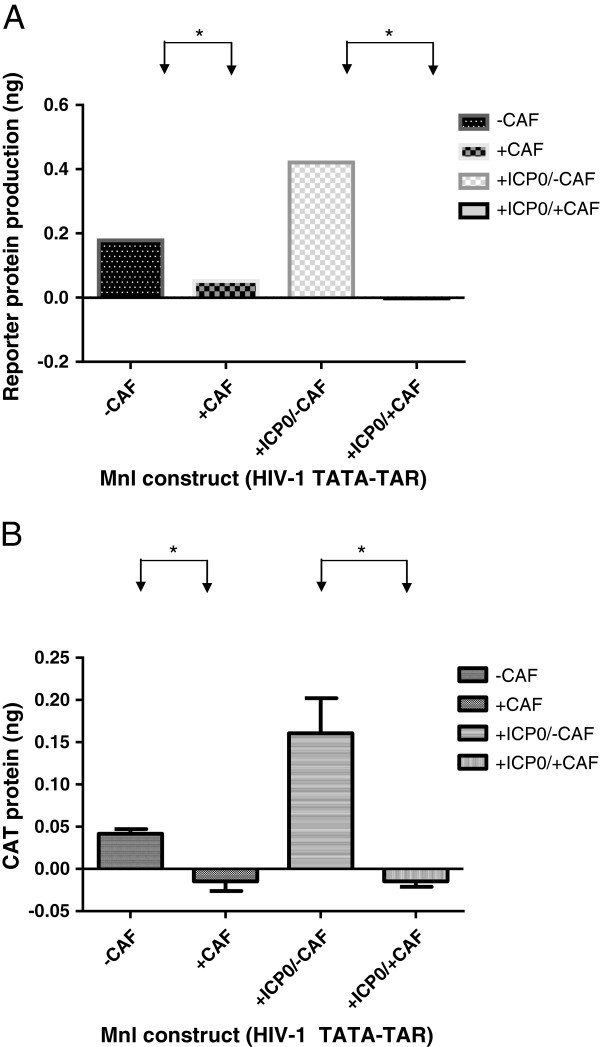
**Transcription of TATA-TAR construct is suppressed by CAF.** Reporter gene product (CAT) expression in cells pretreated with Hanks buffer (-CAF) or conditioned culture medium from a transformed CD8+ T cell line (+CAF) and transfected with a construct containing only the TATA box and TAR from HIV-1 LTR, in the presence or absence of the HSV-1 ICP0 protein. Transcription from the LTR was driven either by CMV-Tat **(Panel A)** or addition of PMA **(Panel B)** Results are representative of 2 independent experiments conducted in triplicate. *, p < 0.05.

Our results with the TAR and SpI deletions/mutations have shown that none of these regions is required for transcriptional suppression. This points towards the CATA box as being important for this process. We attempted to delineate the role of the CATA box by exchanging the CMV TATA box with that of HIV-1 in an HIV-1 LTR construct. We also created a CMV construct with an HIV-1 CATA box, but both of these were transcriptionally inactive and could not be used to assess the ability of CAF to suppress transcription (data not shown). Hence, we used a HIV-1 CATA box-TAR construct (called MnlI, after the restriction enzyme used to create it, see Figure 2) to test this hypothesis. To boost transcription, we used the Herpes virus protein ICP0, which has been shown to increase transcription from the LTR by interacting with Tat protein [34]. We transfected cells with CATA-TAR and ICP0 constructs. Gene expression was induced either with CMV-Tat (Figure 5A) or PMA (Figure 5B). ICP0 was able to increase transcription from the construct, and in the presence of CAF, this transcription was suppressed. Since CAF-mediated suppression was mediated in the absence of the SpI sites, and since TAR [28] was previously found not be required for the suppressive process (as seen in Figure 3B), our results suggest that the region on the HIV-1 LTR necessary for CAF action is likely to reside primarily within or close to the CATA box. Studies have shown that the HIV-1 CATA box regulates the assembly of transcription complexes necessary for processive viral transcription and that these complexes interact with upstream elements for efficient initiation and elongation of transcripts [35]. It is possible that CAF functions by disrupting the complexes necessary for transcription at the CATA box. It is also possible that the upstream Sp1 sites are involved, as mutation of SpI(1) did appear to have an effect on the ability of CAF to completely suppress transcription.

In conclusion, our report shows that CAF can suppress transcription from all regions of the HIV-1 LTR upstream of the CATA box. We did not study epigenetic changes or changes in the CATA-box binding proteins on the HIV-1 LTR in response to CAF. Future work to elucidate the mechanism of CAF in HIV-1 transcription suppression may include these avenues.

## Materials and methods

### Ethical statement

No patients were specifically recruited for the purposes of this study. The CD8+ T-cell line, TG, was previously established by herpesvirus saimiri (HVS)-transformation of CD8+ T cells from a chronically infected HIV-1-infected subject from the Multicenter AIDS Cohort Study (MACS) [19].

### Plasmid constructs

Full length HIV-1 LTR-CAT and the successive deletion constructs at EcoRV, BstNI, HaeIII and MnlI, previously described [27]; the CMV-CAT and CMV-tat constructs; and the ICP0 expression plasmid were generous gifts from Dr. Mario Estable of Ryerson University, Toronto, Canada, Dr. Lung-ji Chang of the University of Florida and Dr. Paul Kinchington, from the University of Pittsburgh, respectively. The BstΔTAR construct was made by digesting the BstNI construct with KpnI and BglII, blunting the ends with Klenow and then ligating the product with T4 DNA ligase. The SV40-luciferase construct was made by PCR-amplifying the SV40 early promoter out from the pSV-beta galactosidase control vector (Promega, WI) using the primers Fwd- ATTAGATCTGCGCAGCACCATG and Rev- CCAGCAGAGATCCCAAGCTTTTT, digesting the PCR product with the enzymes BglII and HindIII followed by ligation to pGL4.10 (Promega, WI) cut with the same enzymes. The CMV-TAR construct was made by amplifying the TAR region out of HIV-1-LTR with the primers F:TTAGGATCCTTTAGTGAACCGGGTCT and R:GCGGATATCTATTGAGGCTTAAGC, digesting the PCR product with the enzymes BamHI and EcoRV and ligating into the CMV-CAT vector cut with the same enzymes. The 3SpI-TATA construct was created by PCR amplifying the SpI-TATA region of the LTR using the primers 5’ATACTCGAGAGGCGTGGCCT and 5’TACAAGCTTCCAGAGAGACCCAGTA, digesting the PCR product with the restriction enzymes XhoI and HindIII, and ligating it to the pGL4.10 plasmid digested with the same enzymes. SpI deletion mutants were created by overlapping PCR, using the specific DNA oligonucleotides and the corresponding primers listed in Table 1. For the point mutations, the mutations introduced to inactivate the SpI sites (italics) are marked in bold, where G nucleotides were substituted by T: ***TT****CGTGGC*CT*G****TT****CGGGAC*T*GG****TT****AGTGGC.* The oligonucleotide pairs and the corresponding primers used to create these constructs are listed in Table 2. For the deletion constructs, each pair of oligonucleotides was added to a PCR mix containing dNTPs, MgCl2 and DNA polymerase; the reaction was cycled without primers for 5 cycles, with these conditions: (94C, 2’30”; [94C-1’ , 55C-1’ , 72C- 1’45”]X5; 72C-10’), the corresponding primers were then added at a final concentration of 10uM and the tube was cycled for 25 cycles at the same cycling conditions. The PCR product was purified, digested with HindIII and SacI and ligated, using T4 DNA ligase, to the BstΔTAR construct cut with the same enzymes. For the point mutations, the primers used were: Fwd: TGTGAAGCTTTCGGAGGACAGTACTC and Rev TGTGGAGCTCGGATCTGGTCTAAC. To make the point mutation constructs, each of the oligo pairs for constructs K- P (see Table 2) was added at a final concentration of 10 mM to a PCR mix with only the reverse primer, at a final concentration of 1uM and the reaction was amplified for 10 cycles at the following conditions: (94C, 2’30”; [94C-1’ , 55C-1’ , 72C- 1’45”]X10; 72C-10’). Following this, the forward primer was added at a final concentration of 1uM, and the reaction mixture was cycled 25 more times at the same conditions. The PCR product was purified, digested with HindIII and SacI and ligated, using T4 DNA ligase, to the BstΔTAR construct cut with the same enzymes. The sequences of all constructs were confirmed by sequencing with the primer 5’CGCTGGGCCCTTCTTAA, present on the luciferase gene or with the primer 5':CAGCTGAACGGTCTGGTTATAG present on the CAT gene. CMV-Renilla luciferase (Promega, Madison, WI) was used as transfection control.

**Table 1:** SpI deletion construct oligos and primers

**S No**	**Name of construct**	**DNA oligos used**
1	Del 1, del 2	5’TCGGAGGACAGTACTCCGACCCGGTCGAAGGGAGGCGTGGCCTGAGCCCTCAGATCCTGCA**TATAA**GC
		And
		5’GAGCCCTCAGATCCTGCA**TATAA**GCAGCTGCTTTTTGCCTGTACTGGGTCTCTCTGGTTAGACCAGATCCGAGC
2	Del 1	5’TCGGAGGACAGTACTCCGACCCGGTCGAAGGGAGGCGTGGCCTGGGCGGGACTGAGCCCTCAGATCCTGCA**TATAA**GC
		And
		5’GAGCCCTCAGATCCTGCA**TATAA**GCAGCTGCTTTTTGCCTGTACTGGGTCTCTCTGGTTAGACCAGATCCGAGC
3	Del 2	5’TCGGAGGACAGTACTCCGACCCGGTCGAAGGGAGGCGTGGCCTTGGGGAGTGGCGAGCCCTCAGATCCTGCA**TATAA**
		And
		5’GAGCCCTCAGATCCTGCA**TATAA**GCAGCTGCTTTTTGCCTGTACTGGGTCTCTCTGGTTAGACCAGATCCGAGC

PCR primers used for amplifying all the constructs: Fwd TGTGAAGCTTTCGGAGGACAGTACTC, Rev: TGTGGAGCTCGGATCTGGTCTAAC.

**Table 2:** SpI inactivating point mutation construct oligos and primers

**3**	**2**	**1**	**Construct**
mut	Wt	wt	K
mut	Wt	mut	L
mut	Mut	wt	M
wt	Mut	wt	N
wt	Mut	mut	O
wt	Wt	mut	P
Construct	Oligos used (Oligo 2 is the same for all constructs)		
K	Oligo 1: TCGGAGGACAGTACTCCGACCCGGTCGAAGGGATTCGTGGCCTGGGCGGGACTGGGGAGTGGCGAGCCCTCAGATCCTGCA**TATAA**		
	Oligo 2: GAGCCCTCAGATCCTGCA**TATAA**GCAGCTGCTTTTTGCCTGTACTGGGTCTCTCTGGTTAGACCAGAT*C*CGAGC		
L	Oligo 1: TCGGAGGACAGTACTCCGACCCGGTCGAAGGGATTCGTGGCCTGGGCGGGACTGGTTAGTGGCGAGCCCTCAGATCCTGCA**TATAA**		
M	Oligo 1: TCGGAGGACAGTACTCCGACCCGGTCGAAGGGATTCGTGGCCTGTTCGGGACTGGGGAGTGGCGAGCCCTCAGATCCTGCA**TATAA**		
N	Oligo 1: TCGGAGGACAGTACTCCGACCCGGTCGAAGGGAGGCGTGGCCTGTTCGGGACTGGGGAGTGGCGAGCCCTCAGATCCTGCA**TATAA**		
O	Oligo 1: TCGGAGGACAGTACTCCGACCCGGTCGAAGGGAGGCGTGGCCTGTTCGGGACTGGTTAGTGGCGAGCCCTCAGATCCTGCA**TATAA**		
P	Oligo 1: TCGGAGGACAGTACTCCGACCCGGTCGAAGGGAGGCGTGGCCTGGGCGGGACTGGTTAGTGGCGAGCCCTCAGATCCTGCA**TATAA**		

### Cells, cell culture and CAF preparations

293 T cells were obtained from the ATCC and cultured in DMEM, supplemented with 10% FBS, 2 mM Glutamine and 1 mM sodium pyruvate. TZM-bl cells were obtained through the NIH AIDS Research and Reference Reagent Program, Division of AIDS, NIAID, NIH: TZM-bl from Dr. John C. Kappes, Dr. Xiaoyun Wu and Tranzyme Inc. The CD8+ T cell line, TG, was previously established by Herpes virus saimiri (HVS)-transformation of CD8+ T cells from a chronically HIV-1 infected subjected from the MACS. TG cells were grown in RPMI with 20% FBS (100 nm filtered, Invitrogen Life Sciences, Carlsbad, CA), supplemented with 25 mM HEPES, Penicillin (100 U/mL) and Streptomycin (100 ug/mL) and rIL2 (50 U/mL, Roche Diagnostics). CAF from these transformed CD8+ T cells was prepared as described before [8]. Briefly, TG cells were cultivated for 14 days, after which, the cells were centrifuged at 300 g and the resulting supernatant was then further centrifuged at 4°C at the following speeds: 2000 g for 30 minutes, 6000 g for 20 minutes and 15000 g for 1 hour to remove other debris. This conditioned media was used for further investigations on CAF.

### Transfections

293 T cells were plated at a concentration of either 200,000 cells/mL in a 6- well plate (if the reporter gene assay was CAT), or 20,000 cells/100 uL in a 96-well plate (if the reporter gene assay was luciferase), in triplicate. 24 hours after plating, 10% vol/vol of CAF from TG cells was added to the culture. 24 hours later, either 1ug or 10 ng (depending on whether the assay was for CAT or luciferase respectively) of the relevant plasmid/s was transfected into all cells, using Lipofectamine Plus (Invitrogen, Carlsbad, CA), according to manufacturer’s instructions, along with the transfection control, CMV-Renilla plasmid. 24 hours following transfection, the cells were stimulated with 100 ng/mL phorbolmyristoylacetate (PMA) for 18 hours or 6 hours (depending of whether the assay was for CAT or luciferase respectively), after which they were lysed and the reporter gene product was measured. Measurement of the reporter gene was done after normalizing for total protein content and Renilla expression. For the experiment detailed in Figure 5, with ICP0 to boost transcription of the Mnl construct, 293 T cells were plated at a density of 200,000/mL in a 6 well plate, in triplicate. 24 hours after plating, 10% vol/vol of CAF from TG cells was added to the culture. 1ug of Mnl-CAT with or without 0.5 ug of ICP0 expression plasmid was transfected, into designated wells. Expression from the Mnl-LTR construct was induced either with co-transfection with CMV-tat expression plasmid (0.5 ug per well) or with PMA (100 ng/mL for 18 hours). CMV-renilla was transfected as a control. 36 hours after transfection, cells were lysed and CAT protein content quantified after normalizing for total protein (as measured by Bradford Assay) and Renilla luciferase levels. CAT and luciferase protein expressions were quantified by CAT ELISA (Roche) and Bright Glo systems (Promega), respectively, according to manufacturer’s instructions. Renilla content was measured using Stop-and-Glo kit (Promega).

## Competing interests

The authors declare that they have no competing interests.

## Authors’ contributions

VS designed the experiments, acquired and analyzed the data and wrote the manuscript; YC designed experiments and wrote the manuscript; PG analyzed the data and wrote the manuscript. All authors read and approved the final manuscript.
